# AHNAK2 is a potential prognostic biomarker in patients with PDAC

**DOI:** 10.18632/oncotarget.15990

**Published:** 2017-03-07

**Authors:** Di Lu, Junxiong Wang, Xiaoyan Shi, Bing Yue, Jianyu Hao

**Affiliations:** ^1^ Department of Gastroenterology, Beijing Chao-Yang Hospital, Capital Medical University, Beijing 100020, China; ^2^ Department of Pathology, Beijing Friendship Hospital, Capital Medical University, Beijing 100050, China

**Keywords:** AHNAK2, pancreatic ductal adenocarcinoma, overall survival, prognostic factor, nomogram

## Abstract

**Background:**

AHNAK nucleoprotein 2 (AHNAK2) belongs to the AHNAK protein family. The studies of AHNAK2 are limited. A recent study reported that AHNAK2 might be a biomarker for pancreatic ductal adenocarcinoma (PDAC); however, tissue-based experiments have not been conducted. The aim of this study was to determine the tissue expression of AHNAK2 and to find the correlation between AHNAK2 and overall survival rate in PDAC.

**Results:**

AHNAK2 is highly expressed in PDAC (n=79) compared with adjacent normal tissues (n=64, *P*<0.001). Overexpression of AHNAK2 showed a significant relationship with a lower overall survival rate (*P*=0.033) in PDAC patients. The predictive value of increased expression of AHNAK2 remains relevant in patients with AJCC grade above II (n=43, *P*=0.006) or lymph node metastasis (n=32, *P*=0.004). Cox regression analysis showed that AHNAK2 expression (*P*=0.003) and pathology grade (*P*<0.001) are independent prognostic factors for PDAC. The nomogram model was performed to predict the 1- and 3-year survival rates based on Cox regression. The C-index was 0.61. The calibration curves were also made to show the association between the observed and predicted probability of the overall survival rates.

**Materials and Methods:**

AHNAK2 expression was performed in tissue microarrays by immunohistochemistry. The overall survival rate analysis was performed using the Kaplan–Meier method, Cox proportional hazards regression, and a nomogram model.

**Conclusions:**

AHNAK2 is overexpressed in PDAC tissues and is an independent prognostic factor in patients with PDAC.

## INTRODUCTION

Pancreatic ductal adenocarcinoma (PDAC), the major pathologic type of pancreatic cancer, is one of the most frequent malignant tumors worldwide [1]. In the United States, the 5-year relative survival rate of PDAC is < 10% due to the low detection rate in the early stage [2]. Moreover, PDAC is sometimes difficult to distinguish from other pancreatic diseases, such as chronic pancreatitis (CP), even when endoscopic ultrasound-guided fine-needle aspiration (EUS-FNA) is performed [3, 4].

It is important to find effective biomarkers to make the diagnosis and predict the prognosis precisely so that we can make optimal decisions for chemotherapy or other adjuvant therapies. However, just carbohydrate antigen 19-9 (CA 19-9) and carcinoembryonic antigen (CEA) have been proved having limited diagnostic and prognostic value for PDAC [5]. New effective biomarkers are needed.

AHNAK nucleoprotein 2 (AHNAK2) gene locating at 14q32 was identified in 2004. AHNAK2 is a large protein (>600 kDa) with a PDZ domain, and it has recently been reported as a potential biomarker of PDAC; however, the previous study did not include tissue-based evidence [6, 7]. In this present study, we first used tissue microarray (TMA) to determine the expression of AHNAK2 in PDAC. Furthermore, we evaluated the prognostic values of AHNAK2 and made a nomogram model to predict the 1- and 3-year overall survival (OS) rates.

## RESULTS

### Clinical characteristics of patients

A total of 79 PDAC patients were included in this study, of which 64 had adjacent normal tissues. Surgery was performed between September 2004 and December 2008 and the patients were followed until October 2012 or death. The median followed time (interval between surgery and the last visit or death) was 12 months (range, 0.6-87 months). Fifty-six patients died due to PDAC during the follow-up period. None of the patients received chemotherapy or radiotherapy before or after surgery. The margin was at least 5mm far from the tumor. The surgeries were all R0 resections. Age, gender, pathologic grade, TNM stage (according to AJCC), family history, smoking history, alcohol consumption history, and type II diabetes history was provided by the documents. Age and gender information only was available for the 45 CP patients.

The patients with PDAC and the adjacent normal tissue (control) cohort did not show any significant difference in baseline characteristics. The CP patients were not compared with the other two groups because of limited detailed information (Table 1).

**Table 1:** Base line characteristics of patients and tumors

Characteristics	Groups			*P* value
	PDAC(n=79)	Control tissue(n=64)	CP(n=45)	
Age	60.8±11.2	60.2±10.6	56.4±8.3	0.75
Gender (Male/female)	47/32	39/25	27/18	0.86
Family history	0	0	NG	>0.99
Smoking history	18 (22.8%)	18 (28.1%)	NG	0.56
Drinking history	17 (21.5%)	18 (28.1%)	NG	0.44
Type II diabetes	13 (16.5)	12 (18.8%)	NG	0.52
Pathology grade				
I-II	57 (72.2%)			
above II	22 (27.8%)			
Vessel/nerve invasion				
Yes	36 (45.6%)			
No	43 (54.4%)			
Tumor invasion depth				
T1, T2	63 (83.5%)			
T3, T4	16 (16.5%)			
Lymph node metastasis				
N0	47 (59.5%)			
N1	32 (40.5%)			
Distant metastasis				
M0	78 (98.7%)			
M1	1 (1.3%)			
AJCC-stage				
I-II	36 (45.6%)			
above II	43 (54.4%)			

Data analysis was only performed between PDAC and control groups.

*P*<0.05 was considered as statistically significant.

Only the PDCA cohort had the baseline information of pathology grade, vessel/nerve invasion, TNM stage and AJCC stage.

### AHNAK2 expression and the correlation with the clinicopathologic features

**Figure 1:**
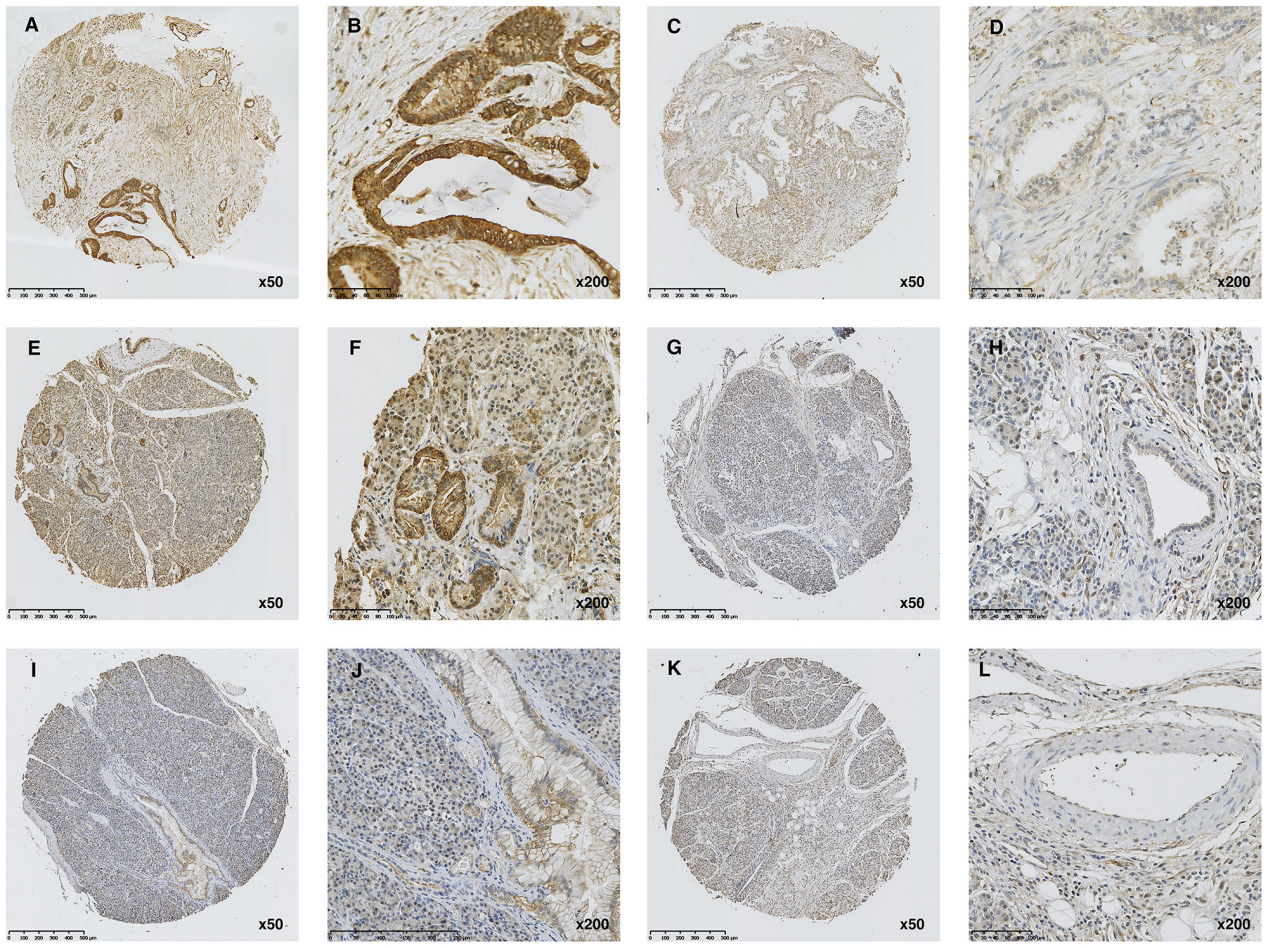
Expression of AHNAK2 in tissue samples **(A-B)** High expression of AHNAK2 in PDAC tissue. **(C-D)** Low expression of AHNAK2 in PDAC tissue. **(E-F)** High expression of AHNAK2 in adjacent normal tissues. **(G-H)** Low expression of AHNAK2 in adjacent normal tissues. **(I-J)** High expression of AHNAK2 in CP tissue. **(K-L)** Low expression of AHNAK2 in CP tissue.

Immunohistochemistry was performed to determine the expression of AHNAK2 in tissue samples. As shown in Figure 1A-1L and Table 2, AHNAK2 was mainly located in the cytoplasm and membranes in epithelial cancer cells and significantly overexpressed in the PDAC group compared with the control group (*P*<0.0001).

**Table 2:** AHNAK2 expression in PDAC and control cohorts

Groups	AHNAK2 expression		*P* value
	High	Low	
PDAC	48 (60.8%)	31	<0.0001
Control	1 (1.6%)	63	
CP	1 (2.2%)	44	

*P*<0.05 was considered as statistically significant.

Data analysis was only performed between PDAC and control groups.

A comparison between AHNAK2 expression and the clinicopathologic features showed that AHNAK2 expression did not correlate with any clinicopathologic features (Table 3).

**Table 3:** Association between AHNAK2 expression and the clinicpathological features of PDAC

Characteristics	Number	AHNAK2 expression		*P* value
		High (n=48)	Low (n=31)	
Age (years)				
<65	49	31	18	0.56
≥65	30	17	13	
Gender				
Male	47	25	22	0.10
Female	32	23	9	
Pathology grade				
I-II	57	35	22	0.85
above II	22	13	9	
Vessel/nerve invasion				
Yes	36	21	15	0.69
No	43	27	16	
Tumor invasion depth				
T1, T2	63	41	22	0.12
T3, T4	16	7	9	
Lymph node metastasis				
N0	47	26	21	0.56
N1	32	22	10	
Distant metastasis				
M0	78	47	31	>0.99
M1	1	1	0	
AJCC-stage				
I-II	36	21	15	0.69
above II	43	27	16	
Family history				
Yes	0	0	0	>0.99
No	79	48	31	
Smoking history				
Yes	18	10	8	0.61
No	61	38	23	
Drinking history				
Yes	18	11	7	0.97
No	61	37	24	
Type II diabetes				
Yes	13	8	5	0.95
No	66	40	26	

*P*<0.05 was considered as statistically significant.

### Prognostic values of AHNAK2 expression

**Figure 2:**
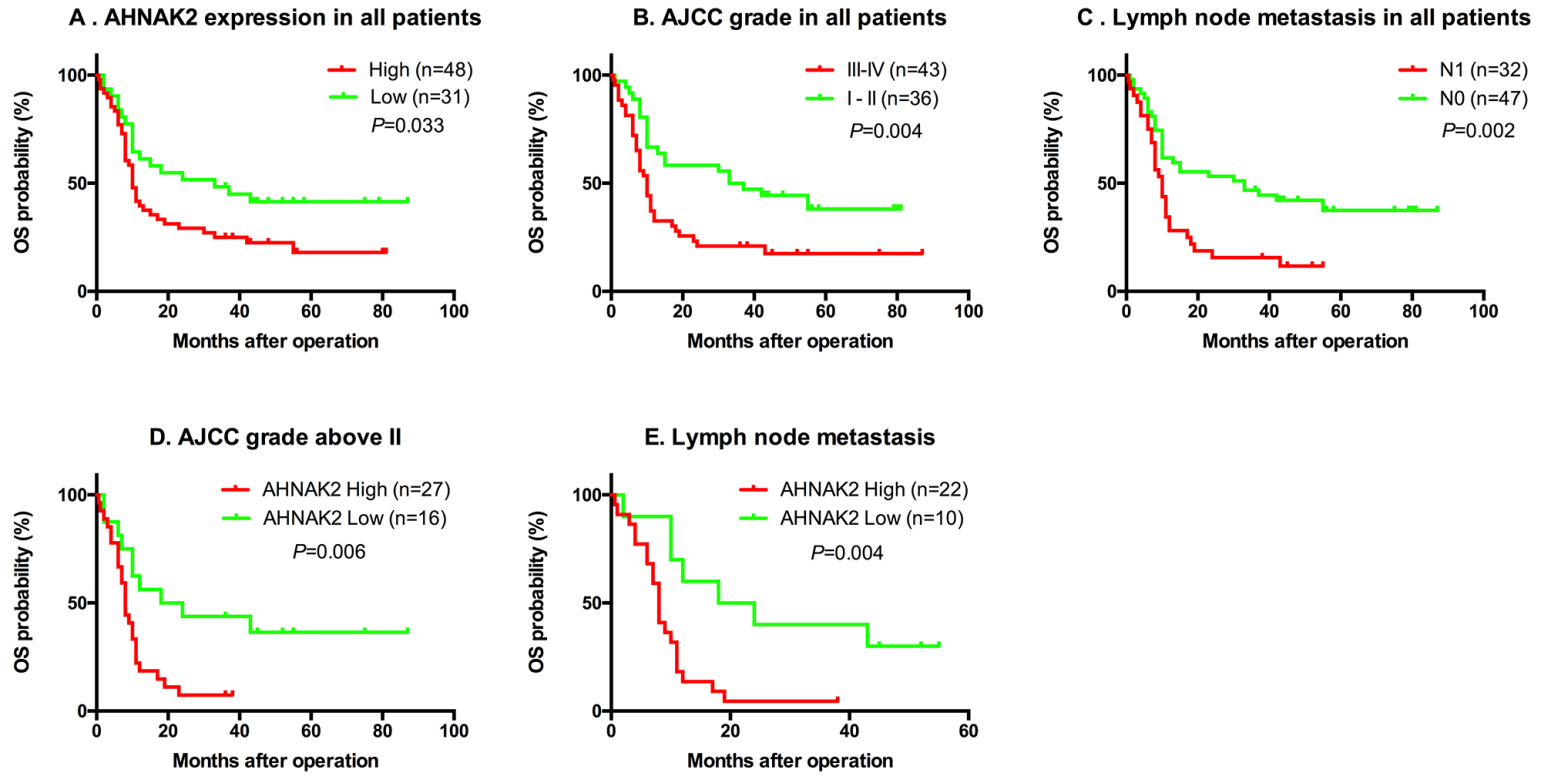
The Kaplan–Meier method analysis with log-rank test for OS **(A-C)** Patients with higher expression of AHNAK2, lymph node metastasis or higher AJCC stage had lower OS. **(D-E)** In the sub-group of AJCC grade above II or lymph node metastasis, the lower OS was significant associated with higher AHNAK2 expression.

To estimate the clinical prognostic significance of AHNAK2 expression, Kaplan–Meier survival analysis and a log-rank test were performed. As shown in Figure 2A-2C, patients with higher expression of AHNAK2, lymph node metastasis, or a higher AJCC stage had a lower OS.

We also determined whether AHNAK2 expression has more prognostic value in advanced cancers or not. As shown in Figure 2D and 2E, in the sub-group of patients with AJCC grade greater than II or lymph node metastasis, a lower OS was significantly associated with higher AHNAK2 expression.

Furthermore, univariate and Cox multivariate regression analyses were used to confirm that AHNAK2 expression and pathologic grade were confirmed to be independent prognostic factors (Table 4).

**Table 4:** Univariate and multivariate analyses of factors associated with survival

Factors	Univariate *P* value	Cox regression	
		HR (95% CI)	*P* value
AHNAK2 expression	**0.033**	**2.627 (1.386-4.978)**	**0.003**
Gender	0.92	0.555 (0.282-1.086)	0.086
Age	0. 96	1.203 (0.664-2.177)	0.542
Pathology grade	0.13	**3.876 (1.840-8.164)**	**<0.001**
Vessel/nerve invasion	0.47	0.603 (0.327-1.113)	0.106
T	0.96	0.959 (0.312-2.945)	0.941
N	**0.002**	4.123 (0.892-19.051)	0.070
AJCC grade	**0.004**	1.342 (0.290-6.220)	0.707
Smoking history	0.43	2.428 (0.896-6.578)	0.081
Drinking history	0.92	0.753 (0.271-2.093)	0.587
Type II diabetes	0.65	0.711 (0.336-1.504)	0.372

*P*<0.05 was considered as statistically significant.

Distant metastasis and family history data was not shown in the table because of the too small number of patients in the sub-groups.

### Nomogram

**Figure 3:**
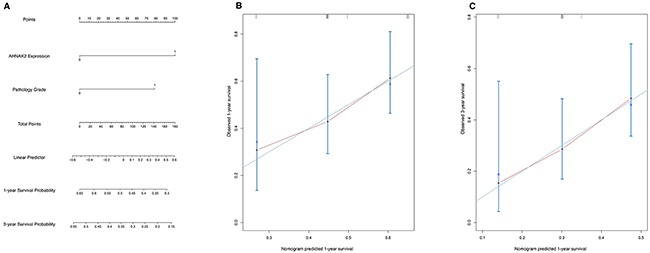
The nomogram and calibration graph **(A)** The nomogram model based on the Cox regression to predict 1-year and 3-year survival rate. The c-index of this model was 0.61. **(B-C)** The calibration graph to show difference between the observed outcome frequencies and the predicted probabilities.

For prediction of the 1- and 3-year OS rates in PDAC patients, AHNAK2 expression and pathologic grade were involved in the nomogram model. The 1- and 3-year survival rates are shown in Figure 3A. The c-index of this model was 0.61. The difference between the observed outcome frequencies and the predicted probabilities are shown in the calibration graph (Figure 3B and 3C).

## DISCUSSION

The AHNAK protein family has 2 members (AHNAK1 and AHNAK2). AHNAK1 was firstly identified in 1992 [8], which has a variety of functions, including membrane repair [9, 10], formation of the blood-brain barrier [11], and regulation of calcium channels [12].

In recent years, a number of researchers have reported that AHNAK1 is differentially expressed and has a variety of functions in different types of cancer, including pancreatic cancer [13], gastric cancer [14], lung cancer [15, 16], breast cancer [17–23], melanoma [24, 25], oral and laryngeal carcinoma [26–28], clear cell renal cell carcinoma [29–31], meningioma [32], and acute lymphocytic leukemia [33].

In those studies, some researchers have demonstrated that AHNAK1 plays an anti-cancer role through epithelial-to-mesenchymal transition (EMT), reorganization of the actin cytoskeleton network, formation of pseudopodial protrusions, or activation of some signaling pathways. In contrast, some other studies have suggested that AHNAK1 is a tumor suppressor by regulating the TGFβ/Smad signaling pathway [34].

Studies which have focused on the function of AHNAK2 are limited. AHNAK2 is a large protein (> 600 kDa), which was first detected in 2004. AHNAK2 was shown to have a similar function to AHNAK1. Moreover, these two AHNAKs may be complementary proteins since knocking down AHNAK1 could lead to the overexpression of AHNAK2 and the mouse has no obvious phenotypes [6].

In a recent study, AHNAK2 was confirmed to be an important element of the FGF1 non-classical export pathway that depletes AHNAK2 leads to a decrease in stress-induced FGF1 export [35]. FGF1 has been identified a key driver of many kinds of solid tumors [36]. AHNAK2 has also been reported to be involved in human esophageal squamous carcinoma (ESCC) by regulating protein lysine mono-methyltransferase SMYD2, which is overexpressed or amplified in various types of cancers [37].

However, AHNAK2 was never been confirmed as a cancer biomarker until Manoj reported that AHNAK2, together with other 4 proteins, could help diagnose PDAC with high sensitivity and specificity; however, there was no tissue-based evidence in that study [7].

Our study first reported that AHNAK2 is highly expressed in PDAC compared to normal tissues by immunohistochemistry. Interestingly, AHNAK2 was highly expressed in all sub-groups of the PDAC cohort. Thus, we suggest that AHNAK2 is a very important molecule in every stage of PDAC.

We also determined the expression of AHNAK2 in CP tissues because it is sometimes difficult to distinguish PDAC from CP [38], even though imaging and endoscopic techniques, such as CT, MRI, and EUS-FNA, have been well-established. The results of immunohistochemistry in CP tissues showed decreased expression of AHNAK2, indicating that AHNAK2 could be used in the differential diagnosis when a CP patient has suspected cancer. Unfortunately, we did not have sufficient data to compare the CP group with the PDAC and control groups. A new cohort with adequate information is needed.

More importantly, we found that AHNAK2 and pathology grade are independent prognostic factors for PDAC after Kaplan–Meier and Cox regression analyses. Furthermore, AHNAK2 had significant predictive ability in sub-groups of patients with AJCC grade greater than II or lymph node metastasis. These findings suggested that AHNAK2 may have a greater role in advanced cancers. In addition, we created a nomogram and calibration graph to predict the 1- and 3-year survival rates. The c-index was 0.61. The nomogram might be useful for predicting patients’ survival time and give them adjuvant therapies.

Our study had some limitations. First, in this retrospective study the patients may have had some selection bias due to the small number of patients, and the documents did not have the disease-free survival, progression-free survival, and time-to-progress. Second, we did not measure the expression of AHNAK2 in serum samples because it was not possible to obtain blood samples. Third, the CP cohort had insufficient clinical baseline information to compare with the PDAC and CP cohorts. Finally, the expression of AHNAK2 and function in PDAC cell lines and related signaling pathways were not investigated.

In conclusion, we showed that AHNAK2 is highly expressed in PDAC and is an independent prognostic factor. Furthermore, we created a nomogram to predict the 1- and 3-year OS. The most important work in the future will be to perform prospective multicenter clinical trials with external validation cohorts with a focus on elaborate cellular and molecular studies.

## MATERIALS AND METHODS

### Patients and tissue microarray (TMA)

The PDAC TMA (diameter, 1.5 mm; 4 μm) constructed by Biochip (Shanghai, China) included 80 primary PDAC samples with adjacent normal tissues and 20 single primary PDAC tissues. After excluding squamous carcinoma, mucous carcinoma, and damaged tissues, 79 PDAC and 64 adjacent normal tissues were available. The pancreatitis TMA (diameter, 1.5 mm; 4 μm) was obtained from US Biomax (Rockville, MD, USA) and included 3 acute pancreatitis (excluded) and 45 chronic pancreatitis samples.

Ethics Committee approval was obtained by Shanghai Biochip and Xi’an Alena-bio Companies that provided the TMAs.

### Immunohistochemistry

The TMAs were deparaffinized, rehydrated, and washed with PBS, followed by antigen retrieval using EDTA (pH 9.0) for 5 min under high pressure. The slides were then incubated with 3 % H_2_O_2_ for 15 min to block endogenous peroxidase. Then, the slides were incubated with rabbit anti-human AHNAK2 polyclonal primary antibody (HPA004145; Sigma-Aldrich, St. Louis, MO, USA) at a 1:500 dilution overnight at 4 °C. After PBS washing, the secondary antibody and DAB (PV8000; ZSGB-BIO, Beijing, China) were added as a chromogen. Finally, the slides were counterstained with hematoxylin. The negative control was performed using pre-immune rabbit serum at the same dilution.

The TMAs were independently scanned by two professional pathologists at ×200 magnification. ImageJ (version 1.50i; National Institutes of Health, Bethesda, MD, USA) was used to measure the percentage of positively-stained epithelial cancer cells, and the percentage was scored as 0 (none), 1 (<10%), 2 (10–50%), 3 (51–80%), and 4 (>80%). The intensity of staining was scored as follows: 0 (no staining); 1 (weak); 2 (moderate); and 3 (strong). The total score was multiplied by the intensity and the percentage score; 0-6 was low expression and 7-12 was high expression [39, 40].

### Statistical analyses

Data analyses were performed using Graphpad Prism 6 Software (La Jolla, CA, USA) and R 3.3.1 software (http://www.R-project.org). The base line and AHNAK2 expression data were evaluated using a t-test and Fishers exact text. OS was calculated using the Kaplan–Meier method with a log-rank test. The Cox proportional hazards regression model was performed for multivariate analyses. The nomogram was based on the Cox model. Furthermore, we developed a calibration curve to compare the relationship between the observed outcomes and the predicted probabilities. *P* < 0.05 was considered statistically significant.

## Abbreviations

AHNAK2: AHNAK Nucleoprotein 2
PDAC: Pancreatic Ductal Carcinoma
CP: Chronic Pancreatitis
CA 19-9: Carbohydrate Antigen 19-9
CEA: Carcinoembryonic Antigen
OS: Overall Survival
DFS: Disease Free Survival
PFS: Progression Free Survival
TTP: Time to Progress
IHC: Immunohistochemistry
TMA: Tissue Microarray
AJCC: American Joint Committee on Cancer
DAB: Diaminobenzidine
PBS: Phosphate Buffered Saline
EDTA: Ethylene Diamine Tetraacetic Acid
